# Effect of ferroelectric BaTiO_3_ particles on the threshold voltage of a smectic A liquid crystal

**DOI:** 10.3762/bjnano.9.76

**Published:** 2018-03-07

**Authors:** Abbas Rahim Imamaliyev, Mahammadali Ahmad Ramazanov, Shirkhan Arastun Humbatov

**Affiliations:** 1 Institute of Physics of Azerbaijan National Academy of Sciences, H. Javid Ave. 131, AZ1143, Baku, Azerbaijan; 2 Baku State University, Z. Khalilov Str. 23, AZ1148, Baku, Azerbaijan

**Keywords:** colloidal systems, dielectric permittivity, ferroelectric BaTiO_3_ particles, smectic A liquid crystals, threshold voltage

## Abstract

The influence of small ferroelectric BaTiO_3_ particles on the planar–homeotropic transition threshold voltage in smectic A liquid crystals consisting of *p*-nitrophenyl *p*-decyloxybenzoate and 4-cyano-4′-pentylbiphenyl were studied by using capacitance–voltage (*C*–*V*) measurements. It was shown that the BaTiO_3_ particles significantly reduce the threshold voltage. The obtained result is explained by two factors: an increase of dielectric anisotropy of the liquid crystals and the formation of a strong electric field near polarized particles of BaTiO_3_. It was shown that the role of the second factor is dominant. The explanations of some features observed in the *C*–*V* characteristics are given.

## Introduction

Interest in liquid crystals (LC) as a unique state of matter arises not only from a scientific point of view but also because of the great practical significance of LCs [1–3]. Flat panel displays based on LC have great advantages such as low power consumption, low cost, simplicity of manufacturing technology and small dimensions [4]. The growing needs in display technology (improved image quality improving, reduced power consumption and size) requires the enhancing exploitation characteristics of used LC. One of the ways to solve this problem is the purposeful synthesis of new LCs with enhanced parameters, while another alternative way is the constructive combination of LC properties with properties of other functional materials. In polymer-dispersed LCs used in flexible displays, small drops of LC are distributed in a polymer medium [5]. In colloidal LC composites, vice versa, micrometer- or submicrometer-sized particles of various nature (e.g., ferromagnetic or ferroelectric) are dispersed in an LC medium. Even a small amount of such particles leads to colossal changes in the properties of LC and causes qualitatively new effects [6–8]. Particularly interesting results were obtained in colloids consisting of nematic LCs and ferroelectric particles. LCs doped with a small quantity of ferroelectric BaTiO_3_ nanoparticles were found to exhibit a nonvolatile electromechanical memory effect in the isotropic phase [9]. Ferroelectric nanoparticles reduce the threshold voltage by enhancing the dielectric anisotropy of the nematic LC and the system becomes sensitive to the sign of the applied electric field [10]. The Fréedericksz transition occurs at least in two stages when the BaTiO_3_ nanoparticles are added to the liquid crystal [11–12].

In colloids of ferroelectric particles in nematic LCs, as a rule, oleic acid is present as a stabilizer, the role of which is to prevent the aggregation of ferroelectric particles. The oleic acid in many cases worsens the materials properties, for example, reduces the clearing point of the liquid crystal [13].

Thanks to the layered structure, smectic LCs have special physical (mechanical, electrical and optical) properties, which opens up wide possibilities for their application in display and non-display applications [14]. If the smectic A LC is used as a matrix in a colloid, then it is not necessary to use a stabilizer, since the high viscosity due to the presence of translational order in one direction almost eliminates the aggregation of particles. On the other hand, the high viscosity of the smectic A LC causes an electro-optical effect with memory, which makes them highly promising when applied in devices for recording and displaying of information. Despite these advantages, the threshold voltage and rise time of the electro-optical effects in smectic A LCs are much higher in comparison with nematic LCs [15]. In this work, the possibility of reducing the planar–homeotropic transition threshold voltage in smectic A LC by doping with submicron ferroelectric particles of BaTiO_3_ is shown.

## Experimental

The smectic A LC is a mixture of *p*-nitrophenyl *p*-decyloxybenzoate and 4-cyano-4′-pentylbiphenyl with a molar ratio of 1:1. The mixture is in the smectic A phase in the temperature range of 32.5–47.0 °C and has positive dielectric anisotropy.

BaTiO_3_ is a ferroelectric material with high spontaneous polarization (0.26 C·m^−2^ at room temperature) [16–17]. In our experiment, the BaTiO_3_ particles with an average size of 500 nm have been used (US Research Nanomaterials Inc.). BaTiO_3_ particles with a size higher than 100 nm have a tetragonal crystal lattice and are ferroelectric [18]. The volume fraction of BaTiO_3_ particles was 0.002. Dispersion of BaTO_3_ particles in a smectic A LC was carried out according to the following procedure: After weighing (balance ADAM PW-124), particles were added to the LC, then the mixture was shaken for 40 min in Ultrasonic Cleaner (NATO CD-4800) at 75 °C (isotropic phase) with subsequent cooling to 35 °C (smectic A phase). The transition to the smectic A phase at 47 °C prevents the aggregation of particles.

The electro-optical and dielectric properties of the LC were measured in an electro-optical cell at 36 °C. The electro-optical cell with layered sandwiched structure consists of two parallel glass plates separated by a 15 μm Teflon spacer. The internal surfaces of glasses were covered by a transparent and conductive ITO layer. Polyimide was used as a planar aligning material. The process of filling of the electro-optical cell was carried out by a capillary method in the isotropic state of the LC.

The capacitance of the electro-optical cell was measured using an RLC-meter E7-20. The amplitude and frequency of AC test signal were 0.5 V and 1 kHz, respectively. The RLC-meter allows for applying a DC bias voltage of up to 40 V. The threshold voltage (*U*_th_) was determined from the capacitance–voltage characteristics of the electro-optical cell. The electro-optical effect changes the effective dielectric permittivity of LC, which is reflected a change of capacitance of the electro-optical cell. The electro-optical effect in the investigated LC is the planar–homeotropic transition due to the positive dielectric anisotropy of the LC. The threshold voltage is defined as the voltage at which the capacitance starts to increase.

## Results and Discussion

**Figure 1:**
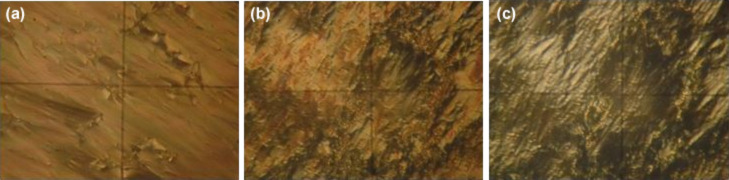
Polarizing microscopy images representing the planar–homeotropic transition in pure smectic A LC: a) *U* = 25 V; b) *U* = 35 V; c) *U* = 45 V. The frame height is 80 μm.

**Figure 2:**
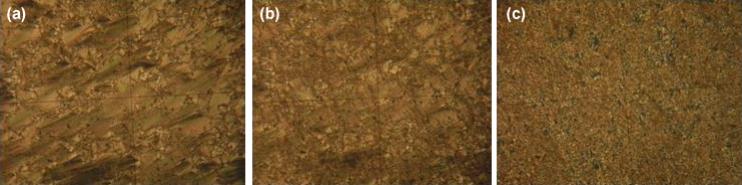
Polarizing microscopy images representing the planar–homeotropic transition in a colloid of smectic A LC + BaTiO_3_: a) *U* = 15 V; b) *U* = 20 V; c) *U* = 25 V. The frame height is 80 μm.

The homeotropic-planar transition in a smectic A LC occurs in the form of deformation, destruction and rearrangement of smectic layers under the action of an electric field. Microphotographs (Figure 1 and Figure 2) present the final pictures after a long-term application of voltage. If the applied voltage exceeds the threshold voltage, the smectic layers are destroyed and rearranged, but an ideal homeotropic structure is not obtained. However, if the applied voltage is sufficiently high, the final state is close to homeotropic.

**Figure 3:**
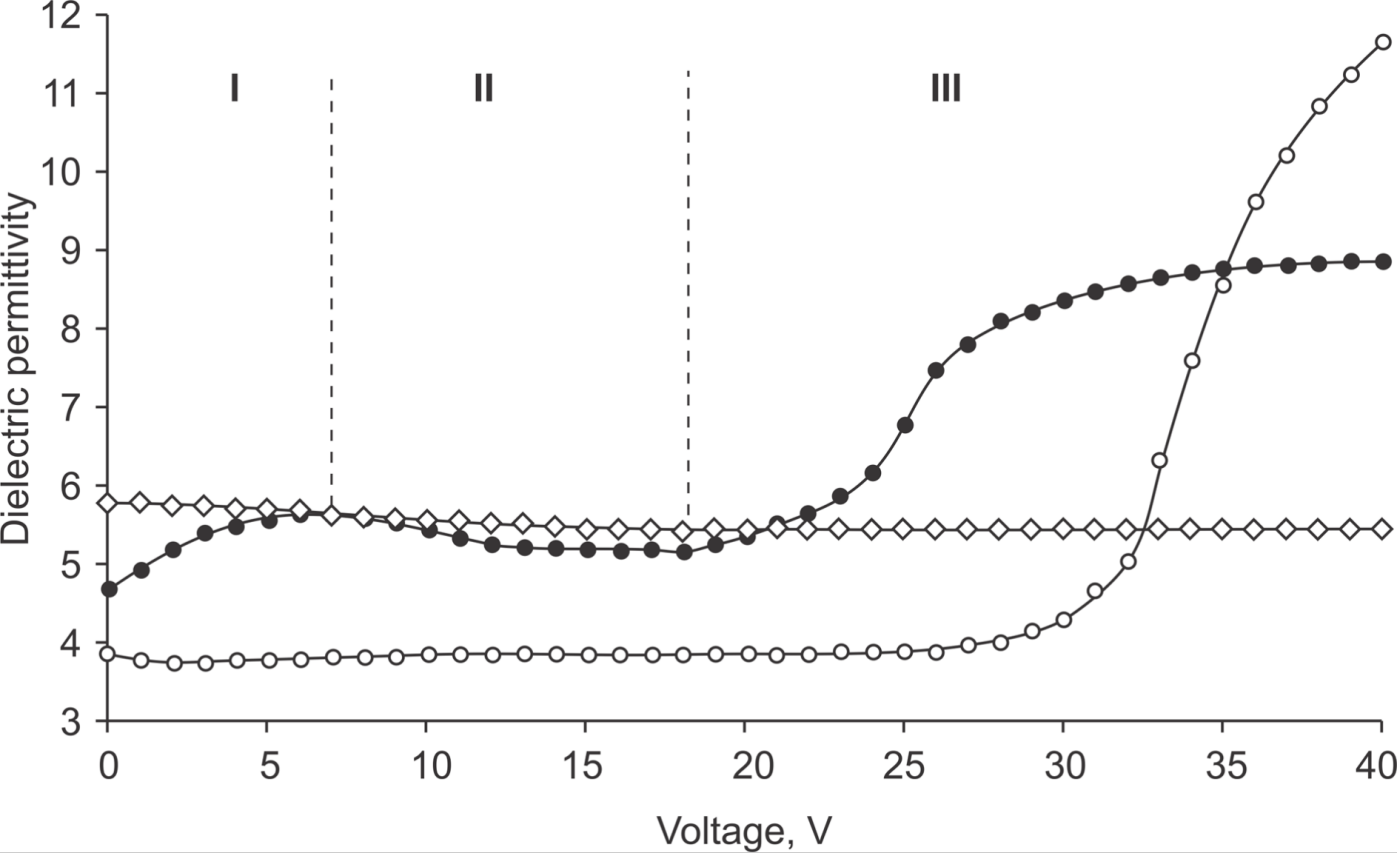
Voltage dependence of effective dielectric permittivity. Circles: pure smectic A LC; filled circles: smectic A LC + BaTiO_3_ colloid in the smectic A phase; diamonds: smectic A LC + BaTiO_3_ colloid in the crystal phase.

As mentioned above, the threshold voltage is determined from the capacitance–voltage measurements of the electro-optical cell initially having planar alignment. The threshold voltage corresponds to the voltage at which the capacitance of the cell or the effective dielectric permittivity starts to increase. Note that the latter is determined as the ratio ε = *C*/*C*_0_, where *C* and *C*_0_ are the capacitances of the filled cell and an empty cell, respectively. Figure 3 presents the effective dielectric permittivity as a function of the voltage for pure smectic A LC (circles) and a colloid of smectic A LC + BaTiO_3_ particles (filled circles). The threshold voltage (*U*_th_ = 18 V) of the colloid is almost one and a half times less than that of pure smectic A LC (*U*_th_ = 28 V). In addition, the colloid exhibits a slight increase (between 0 and 6 V) with a subsequent decrease (between 6 and 18 V) of the permittivity.

The sharp decrease in the threshold voltage with the addition of barium titanate particles can be due to two reasons: 1) an increase in the dielectric anisotropy; 2) the formation of a strong local field around the polarized BaTiO_3_ particles. The BaTiO_3_ particles increase the dielectric anisotropy of a smectic A LC by 1.2-times. The dielectric anisotropy of pure smectic A LC and the colloid measured at a frequency of 1 kHz and amplitude of 0.5 V gives Δε^LC^ = 
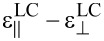
 = 17.0 − 3.6 = 13.4 and Δε^c^ = 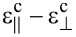 = 19.6 − 4.0 =15.6. In according to the Parodi model of planar–homeotropic transitions in smectic A LCs [15,19], the threshold voltage is determined by the relation:

[1]
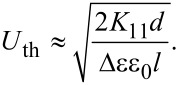


Here, *d* is the cell thickness, *l* is the distance between smectic layers, *K*_11_ is the splay elastic constant and ε_0_ = 8.85 pF/m is the vacuum permittivity. According to [20], the BaTiO_3_ particles practically do not change the value of the elastic constant *K*_11_. Equation 1 shows that the increase in dielectric anisotropy by 1.2 times reduces the threshold voltage only by 1.1-times. In recent theoretical papers devoted to LC colloids [21–22] containing ferroelectric nanoparticles, the reducing of Fréedericksz effect threshold voltage was related to the modification of dielectric anisotropy of LC and the calculated decrease of the threshold was also insignificant.

The strong decrease in the threshold voltage by adding of ferroelectric BaTiO_3_ particles can be explained by the appearance of a strong local electric field around the polarized particles. At a voltage of 15 V and a cell thickness of 15 μm the electric field has the order *E*_0_ ≈ 10^6^ V/m. Such values of the electric field polarize the BaTiO_3_ particles, i.e., switch them from a polydomain to a single-domain state [14]. Polarized particles create a local electric field,


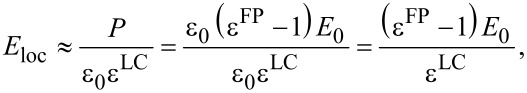


near their surface with ε^FP^ being the dielectric permittivity of the BaTiO_3_ particles. Taking into account that ε^LC^, ε^FP^ and *E*_0_ are of the order of 10, 10^3^ and 10^6^ V/m, respectively, we obtain *E*_loc_ ≈ 10^8^ V/m. Such a strong field around polarized BaTiO_3_ particles is able to cause a planar–homeotropic transition in a smectic A LC. Thus, BaTiO_3_ particles serve as centers from which the planar–homeotropic transition begins at relatively low voltages.

The observation of the weak maximum at 6 V in the case of the colloid (Figure 3) can be explained as follows: In the linear approximation, the dielectric constant of the colloid consisting of liquid crystal and ferroelectric particles can be represented in the form

[2]
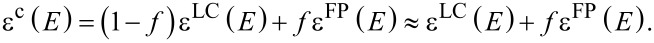


Here, *f* is the volume fraction of ferroelectric particles, ε^LC^(*E*) and ε^FP^(*E*) are the permittivity values of LC and ferroelectric particles both depending on the electric field strength *E*. Ferroelectric particles have a dipole moment due to residual polarization and in the absence of a field their dipole moments are oriented parallel to the LC molecules (polarization–director coupling [23]). When an electric field is applied, these dipoles rotate to oriente themselves in the direction of the field, that leads to an increase in the dielectric constant of the colloid (the first section of the curve in Figure 3). If the applied field exceeds the threshold value, ε^LC^(*E*) significantly increases, which is reflected in the dielectric permittivity of the colloid (third segment). Figure 3 shows that the dielectric constant of the colloid after the planar–homeotropic transition is much lower than that of the pure LC. This is due to the imperfection of the final homeotropic state (Figure 2,c).

The decrease in the permittivity in the voltage range from 6 to 16 V (the second part of the curve in Figure 3) is associated with a slowing of the ratio *P*/*E* when the polarization approaches saturation. According to the formula


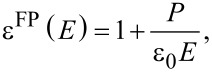


this leads to a decrease in the dielectric permittivity of the particles and the colloid as a whole. This is confirmed by similar measurements in the crystalline phase of the colloid, where any structural changes (the rotation of the particles and the planar–homeotropic transition) are impossible (Figure 3).

## Conclusion

Embedding a small amount of submicrometer-sized ferroelectric BaTiO_3_ particles in a smectic A liquid crystal caused appreciable changes of the electric field dependence of the dielectric constant: An increase in the dielectric constant at small fields is observed, which is explained by the rotation of ferroelectric particles in the LC medium. After that a small decrease due to monodomainization of these particles occurs, and finally a strong increase in the dielectric constant as a result of the planar–homeotropic transition is measured. The doping of smectic A liquid crystal with ferroelectric BaTiO_3_ particles also reduces the threshold voltage of the planar–homeotropic transition, which can be explained by the appearance of a strong local electric field around the polarized BaTiO_3_ particles.
